# Phenotypic and Genomic Insights into Biofilm Formation in Antibiotic-Resistant Clinical Coagulase-Negative *Staphylococcus* Species from South Africa

**DOI:** 10.3390/genes14010104

**Published:** 2022-12-29

**Authors:** Jonathan Asante, Akebe L. K. Abia, Daniel Anokwah, Bakoena A. Hetsa, Dorcas O. Fatoba, Linda A. Bester, Daniel G. Amoako

**Affiliations:** 1 School of Pharmacy and Pharmaceutical Sciences, University of Cape Coast, Cape Coast, Ghana; 2 College of Health Sciences, University of KwaZulu-Natal, Durban 4000, South Africa; 3 Environmental Research Foundation, Westville 3630, South Africa; 4 Biomedical Resource Unit, College of Health Sciences, University of KwaZulu-Natal, Durban 4000, South Africa

**Keywords:** biofilm, adhesion, coagulase-negative staphylococci, bacteria, antibiotic resistance

## Abstract

The work aims to investigate biofilm formation and biofilm/adhesion-encoding genes in coagulase-negative staphylococci (CoNS) species recovered from blood culture isolates. Eighty-nine clinical CoNS were confirmed using the VITEK 2 system, and antibiotic susceptibility testing of isolates was conducted using the Kirby-Bauer disk diffusion method against a panel of 20 antibiotics. Isolates were qualitatively screened using the Congo red agar medium. Quantitative assays were performed on microtiter plates, where the absorbances of the solubilised biofilms were recorded as optical densities and quantified. In all, 12.4% of the isolates were strong biofilm formers, 68.5% had moderate biofilm capacity, and 17.9% showed weak capacity. A subset of 18 isolates, mainly methicillin-resistant *S. epidermidis*, were investigated for adherence-related genes using whole-genome sequencing and bioinformatics analysis. The highest antibiotic resistance rates for strongly adherent isolates were observed against penicillin (100%) and cefoxitin (81.8%), but the isolates showed no resistance to linezolid (0.0%) and tigecycline (0.0%). The *ica*ABC genes involved in biofilm formation were detected in 50% of the screened isolates. Other adherence-related genes, including autolysin gene *atl* (88.8%), elastin binding protein gene *ebp* (94.4%), cell wall-associated fibronectin-binding protein gene *ebh* (66.7%), clumping factor A gene *clf*A (5.5%), and pili gene *ebp*C (22.2%) were also found. The insertion sequence IS256, involved in biofilm formation, was found in 10/18 (55.5%) screened isolates. We demonstrate a high prevalence of biofilm-forming coagulase-negative staphylococci associated with various resistance phenotypes and a substantial agreement between the possession of biofilm-associated genes and the biofilm phenotype.

## 1. Introduction

Coagulase-negative staphylococci (CoNS) have been implicated in myriad infections, including urinary tract infections, bloodstream infections, and endocarditis [1]. *S. epidermidis*, among the CoNS, is the most frequently isolated from biofilm-related infections [2]. Biofilm formation is a critical virulence mechanism utilized by coagulase-negative staphylococci (CoNS) [1,3] that also enables them to survive under harsh conditions, including the presence of antimicrobial agents. Biofilms compromise antibiotic treatment due to their ability to protect bacteria against antibiotics by creating a barrier around the bacterial cell and are estimated to be associated with two-thirds of hospital-acquired infections. In addition, they have been found to possess antibiotic-inactivating enzymes, including β-lactamases, thus creating antimicrobial resistance islands [4]. Biofilms consist of bacterial communities encased in a matrix. The polymer matrix of biofilms reduces the penetration of antibiotics, a process augmented by increasing the biofilm thickness. Furthermore, the electrostatic charges on the polymeric surface of the biofilm bind to charged antimicrobials, reducing their effective concentration [5]. Biofilm formation in bacteria fulfils the dual role of aiding the bacteria in adhering to biotic and abiotic surfaces and helping the bacteria to evade antibiotics and host defence mechanisms [6].

Biofilms can form on biotic surfaces such as host tissue, plasma, or extracellular matrices, or on abiotic surfaces such as inserted medical devices, where they most likely colonise devices during the insertion period after they have been exposed to patients’ skin and mucous membrane [2]. Thus, they can persist and be sustained at infection sites and beyond. The polysaccharide intercellular adherence (PIA)-producing component known as the ica *(icaADBC*) operon, along with the microbial surface components recognizing adhesive matrix molecules (MSCRAMMs), a class of staphylococcal virulence factors that facilitate the adherence of staphylococci to components of the host extracellular matrix, play a part in the formation of biofilm [7,8].

Biofilm formation can be studied by using phenotypic techniques that investigate the abilities of strains to produce an extracellular polysaccharide matrix or their growth onto a surface. For each these principles, different methods have been advanced [9,10,11]. Additionally, biofilms in staphylococci can be investigated genotypically by investigating the genes involved in their formation.

The first step in biofilm formation involves the adherence of bacterial cells to a surface. This is followed by the aggregation of cells [12]. Other genetic determinants of biofilm formation include the *fnb*A and *fnb*B genes, which encode the fibrinogen-binding proteins A and B, respectively [13]; *bap*, which encodes the biofilm-associated protein [14]; *aap*, encoding the accumulation-associated protein [15]; and *embp*, encoding the extracellular matrix binding protein [16]. In addition, adherence determinants include the autolysin gene (*atl*), cell wall-associated fibronectin-binding protein gene (*ebh*), elastin binding protein gene (*ebp*), Ebp pili (*ebp*C), Ser-Asp rich fibrinogen-binding protein genes (*sdr*G and *sdr*H), fibronectin-binding proteins (*pav*A), D-alanine-polyphosphoribitol ligase (*dlt*A), and clumping factor A gene (*clf*A) [17]. The *atl* gene, for instance, encodes the protein that exhibits vitronectin-binding activity and is involved in the primary attachment of *S. epidermidis* to a polystyrene surface, and is similar to the major autolysin of *S. aureus* [18].

Approaches for the control of biofilms involve three main techniques: (i) reduction of planktonic cells before they can form biofilms, (ii) initial inhibition of adherence to surfaces, and (iii) the removal or disruption of formed mature biofilms [19]. As bacteria in a biofilm are more resistant to antimicrobial agents [20], the focus of therapies has generally been on preventing biofilm formation [21]. However, the mechanisms by which biofilms contribute to bacterial persistence in hospital environments are still not fully understood and are still subjects of study. Understanding biofilm formation is essential to inform clinical therapy and hospital infection control. Thus, this study aimed to assess the biofilm-forming ability of CoNS isolates recovered from blood culture isolates from clinical sources in the KwaZulu-Natal Province of South Africa to gain insights into their genetic bases using whole-genome sequencing and bioinformatics analysis.

## 2. Materials and Methods

### 2.1. Bacterial Isolates and Antibiotic Susceptibility Testing

The processes of bacterial isolation, identification, initial characterization, and antibiotic susceptibility testing have been described in a previous study [22]. Briefly, 89 suspected CoNS blood culture isolates were collected from the microbiology units of 3 hospitals from the uMgungundlovu District in the KwaZulu Natal Province in South Africa. Antibiotic susceptibility and molecular confirmation of methicicillin resistance were performed as described in the previous study [22].

### 2.2. Qualitative Biofilm Testing: The Congo Red Assay (CRA) Method

The Congo red agar method previously described [10] was used for qualitative biofilm testing. Briefly, this method is a direct and non-quantitative approach that allows for the identification and differentiation of biofilm-forming microorganisms (black colonies) from non-biofilm-forming strains (red colonies) [23]. The Congo red agar medium was prepared by mixing 37 g of brain heart infusion agar, 5% *w*/*v* sucrose (50 g/L), and 0.08% *w*/*v* (0.8 g/L) Congo red dye. The brain heart infusion (BHI) agar and the sucrose were prepared together, while the Congo red dye was separately prepared as a stock solution and autoclaved at 121 °C for 15 min. The Congo red dye solution was then added to the BHI agar after both solutions had cooled to about 55 °C and allowed to set. The CRA plates were inoculated with one or more colonies of the CoNS isolates and incubated aerobically for 24 h at 37 °C. After incubation, the formation of black colonies was considered positive for biofilm formation, while the formation of pink/red colonies was considered negative for biofilm formation. Brown colonies were considered moderate biofilm formers. *Staphylococcus epidermidis* ATCC 35984 was used as a positive control for strong biofilm formation.

### 2.3. Quantitative Biofilm Assay: Tissue Culture Plate Method

The tissue culture plate assay [24] was used with modifications. Briefly, isolates were grown in trypticase soy broth (TSB) containing 1% glucose at 37 °C for 24 h. Broth cultures of bacteria were diluted 1:100 with a fresh TSB medium. Sterile round-bottom 96-well microtiter plates were inoculated with 200 µL of the bacterial suspension adjusted to 0.5 MacFarland standard and incubated at 37 °C for 24 h without shaking. Uninoculated broth was added as a negative control, while *S. epidermidis* ATCC 35984 was used as a positive control. After incubation, the culture supernatant was discarded. The plates were gently submerged in tap water to wash off remaining unbound cells and medium components that might cause background staining. The plates were washed thrice and allowed to dry at room temperature. Once dry, all wells were stained with 0.1% crystal violet solution and incubated at room temperature for 10 min. Plates were washed three times with distilled water described previously and dried to remove excess liquid. Wells were destained with 125 µL of 30% acetic acid solution, including a blank well with only acetic acid, and incubated at room temperature for 10 min to solubilize the crystal violet retained by the biofilm. The optical density of each sample was measured to quantify the absorbance of biofilm at 570 nm using a microtiter plate reader (BMG LABTECH, Offenburg, Germany). The biofilm formation of each isolate was evaluated in triplicate [24].

The absorbance values were averaged and interpreted as biofilm formation. The formula for classification grouped isolates into three categories based on the optical density (OD) at 570 nm, as follows: OD < ODC = no biofilm producer, ODC < OD ≤ (2 × ODC) = weak biofilm producer, (2 ODC) < OD ≤ (4 × ODC) = moderate biofilm producer and (4 × ODC) < OD = strong biofilm producer, where ODC is the average OD of the negative control. The relative biofilm capacity to the average value of isolates was calculated by the expression

(1) $$=\left[Ax-Ao\right]/\left[\sum_{n=1}^{89}\left(An-Ao\right)/89\right]$$

where *Ax* = the absorbance for isolate *x* at 570 nm and *Ao* = the absorbance for the uninoculated medium [25].

### 2.4. DNA Isolation, Whole-Genome Sequencing, and Bioinformatic Analyses

A sub-sample of 18 methicillin-resistant CoNS (MRCoNS) isolates was selected for whole-genome sequencing (WGS) and screened for genes encoding adherence/biofilm formation. Isolates selected for WGS included 16 *S. epidermidis* isolates and 2 *S. haemolyticus*. Isolates were mainly chosen because of their (mainly *S. epidermidis*) well-documented ability to form biofilms [2] and their resistance to multiple antibiotics (Supplementary Materials). The genomic DNA of the selected isolates was extracted from overnight cultures using the GenElute Bacterial Genomic DNA kit (Sigma Aldrich, St. Louis, MO, USA), according to the manufacturer’s instructions. The DNA was quality-checked and quantified using the Nanodrop™ 1000 spectrophotometer (Thermo Scientific, Wilmington, DE, USA). The Nextera XT DNA Sample Preparation Kit (Illumina, San Diego, CA, USA) was used to prepare the genomic DNA libraries and sequenced on the Illumina MiSeq platform (Illumina, San Diego, CA, USA) at the Sequencing Core Facility, National Institute for Communicable Disease, Johannesburg, South Africa. The raw sequence reads were quality trimmed using Sickle version 1.33 “https://github.com/najoshi/sickle (accessed on 14 September 2022)”, while SPAdes version 3.11 [26] and the CLC Genomics Workbench version 10.1 (CLC, Bio-QIAGEN, Aarhus, Denmark) were used to assemble the reads.

The de novo assembled genomes of sequenced CoNS isolates were queried in relevant databases to detect genetic elements of interest. The Center for Genomic Epidemiology’s KmerFinder “https://cge.cbs.dtu.dk/services/KmerFinder/ (accessed on 17 September 2022)” and the Pathogenwatch platform “https://pathogen.watch (accessed on 17 September 2022)” were used to confirm the identities of isolates and observed phenotypic resistance. To identify genes involved in biofilm/adherence, we used the virulence factor database (VFDB) “http://www.mgc.ac.cn/cgi-bin/VFs/v5/main.cgi?func = VFanalyzer (accessed on 22 September 2022)”, BacWGSTdb “http://bacdb.cn/BacWGSTdb (accessed on 22 September 2022)” and VirulenceFinder 2.0 (using a minimum length of 60% and a threshold of 90%). 

The MLST 2.0 program software version 2.0.9 “https://cge.cbs.dtu.dk/services/MLST/ accessed on 27 September 2022)” and PubMLST “https://pubmlst.org/ (accessed on 27 September 2022)” were used to perform in silico multilocus sequence typing (MLST). First, the internal fragments of the seven housekeeping genes (*arc*C, *aro*E, *gtr*, *mut*S, *pyr*R, *tpi*A, and *yqi*L) were matched to identify alleles to assign sequence types (STs).

The nucleotide sequences of the 18 isolates (C7, C31, C35, C36, C38, C40, C68, C81, C119, C122, C127, C133, C135, C137, C138, C145, C146, and C148) that were whole-genome sequenced were uploaded to the GenBank database under the Bioproject number PRJNA667485.

## 3. Results

### 3.1. Qualitative Biofilm (Congo Red Assay) Method

In total, 35 (39.3%) isolates were positive for biofilm formation based on colour changes to the Congo red agar (CRA) medium, and 50 (56.2%) isolates formed brown colonies indicative of moderate biofilm formation, with 3 (3.4%) considered non-biofilm formers based on the formation of red/pink colonies on the CRA medium (Figure S1, Supplementary Materials). 

Of the 35 isolates determined to be biofilm formers by this method, *S. epidermidis* constituted 11 (31.4%), while *S. hominis* ssp. *hominis*, *S. lentus,* and *S. xylosus* constituted 8 (22.9%), 2 (5.7%), and 3 (8.6%) of the isolates respectively. Table S1 (Supplementary Materials) describes the CoNS species and the antibiotic resistance profiles of the isolates studied.

### 3.2. Tissue Culture Plate Method (Quantitative Method)

Overall, 11 (12.4%) isolates were classified as strong biofilm producers (0.416 < OD), 61 (68.5%) as moderate (0.208) < OD ≤ (0.416), 16 (17.9%) as weak (0.104 < OD ≤ 0.208), and 2 (2.2%) as negative (OD < 0.104), where OD is the average optical density of each sample at 570 nm interpreted as biofilm formation. Table 1 describes the categories of biofilm formation according to the CoNS species obtained. Table S2 (Supplementary Materials) describes the CRA characteristics and classifies the biofilm-forming abilities of CoNS isolates using mathematical formulas. Generally, there was a high level of agreement between the qualitative Congo red assay and the quantitative tissue culture plate method, as most of the black/brown colonies on CRA were classified as either strong, moderate, or weak biofilm formers.

### 3.3. Detection of Biofilm/Adherence-Associated Genes and Sequence Types (STs) Using WGS

The tested isolates were positive for several genes involved in biofilm and adherence formation. The *ica*A, *ica*B, *ica*C genes encoding polysaccharide intercellular adhesin were detected in 9/18 of the screened isolates. We also found the *ica*R gene in six (6) isolates. Other adherence genes detected include the autolysin gene *atl*, elastin binding protein gene *ebp*, cell wall-associated fibronectin-binding protein gene *ebh*, clumping factor A gene *clf*A, Ser-Asp rich fibrinogen-binding proteins *sdr*C, *sdr*G, *sdr*H, *sdr*E, pili gene *ebp*C, fibronectin-binding proteins gene *pav*A, and the polar flagella gene *flm*H (Table 2).

The insertion sequence element IS256, linked to biofilm formation, was detected in 10/18 of the isolates sequenced. Six of the nine *ica*-positive isolates possessed IS256, while four *ica*-negative isolates possessed IS256. We identified eight different MLST types, namely, sequence types (ST) ST2 (two), ST3 (one), ST54 (two), ST59 (two), ST83 (one), ST210 (one), ST490 (one), and ST640 (one). Both ST2 isolates possessed the *ica* genes and IS256. Similarly, both ST54 isolates possessed the *ica* genes and IS256, while the two ST59 isolates were *ica* gene negative. 

## 4. Discussion

The organisation of cells into biofilms compromises the ability of antimicrobials to penetrate the bacterial cells, preventing the build-up of lethal antibiotic concentrations [27]. The clinical relevance of biofilms is related to the fact that an estimated two-thirds of hospital-acquired infections are caused by biofilm-forming bacteria [28]. In this study, we employed phenotypic (qualitative and quantitative) methods, together with whole-genome investigation, to gain insights into the genetic basis of biofilm formation.

*S. epidermidis* (3), *S. hominis* ssp. *hominis* (2), and *S. haemolyticus* (2) were the most abundant species of strong biofilm formers (tissue culture plate method). This observation is consistent with literature that found that antibiotic-resistant S. epidermidis and *S. haemolyticus* strains frequently form biofilms and are responsible for resistant infections, especially among neonates [29]. The abundance of these species on body surfaces, particularly the axillae, inguinal, and perineal areas (*S. epidermidis*), and pubic areas high in apocrine glands (*S. haemolyticus* and *S. hominis*) gives them access to blood during surgical procedures or when there is a break in the skin [1,2]. *S. epidermidis*, among the CoNS, is the most frequently isolated from biofilm-related infections [2]. It is also the most commonly isolated from healthcare-associated infections, especially cardiovascular infections and catheter-related bactaeremia [1]. *S. epidermidis* form biofilms on medical devices and on biotic surfaces that can lead to the breakaway of single cells, spreading and colonising other parts of the body and leading to infections such as endocarditis and sepsis. Thus, strains with biofilm-forming ability are considered more virulent [2]. The ability of the CoNS, particularly the *S. epidermidis* group, to form biofilms is strongly suggestive of selective processes expedited by a modern medical procedure, such as the reliance on antibiotics and the insertion of foreign body devices [2].

Among the 11 isolates classified as strong biofilm producers, 9 (81.8%) were methicillin-resistant by the phenotypic cefoxitin disc diffusion test and PCR detection of the *mec*A gene. Moreover, the isolates exhibiting strong adherence showed high susceptibilities against tigecycline (100%), linezolid (100%), gentamicin (100%), teicoplanin (90.9%), and vancomycin (90.9%), while displaying high resistance against penicillin G (100%) and sulphamethoxazole/trimethoprim (72.7%).

The ica operon, which facilitates the adherence of staphylococci to components of the host extracellular matrix, plays a part in biofilm formation [8]. Furthermore, CoNS, especially *S. epidermidis,* possesses other determinants that facilitate attachment to surfaces and promote various biofilm formation stages. The percentage of positive results for the *ica* (*ica*A, *ica*B, and *ica*C) genes in screened isolates was 9/18 (50%), higher than that recorded in a study in Poland (6.9%) [27]. The *ica*D gene, encoding a helper protein involved in PIA biosynthesis, was not detected in the sequenced isolates. Concerning the *ica* genes, evidence has been put forward to suggest their role in *S. epidermidis* infections, as shown by the prevalence of *ica*-positive strains in blood cultures and mucosal isolates [30]. 

Based on alternating insertion and excision of the insertion sequence element IS256, the IS256 has been closely linked to biofilm formation in pathogenic methicillin-resistant *S. epidermidis* [31]. This assertion was supported by the observation that six out of the ten *ica*-positive isolates possessed IS256. The ica operon is a key factor in the second step of biofilm formation, which is the segregation stage [12]. Six of the nine ica-positive isolates were moderate biofilm formers, while one was a strong biofilm former as determined by the phenotypic tissue culture plate method. In addition, it was observed that some ica-possessing isolates showed weak adherence, while some *ica*-negative isolates showed strong adherence. This unpredictable association between *ica* gene possession and adherence formation in *Staphylococcus* is due to the fact that the expression of biofilm-associated genes and adherence to surfaces is a complex process involving gene regulation and other factors such as pH, nutrients, and surface characteristics [32].

Aside from the *ica*ACDB genes, other genes involved in adherence formation were also detected in isolates subjected to WGS. Such adherence determinants include the autolysin gene (*atl*), cell wall-associated fibronectin-binding protein gene (*ebh*), elastin binding protein gene (*ebp*), Ebp pili (*ebp*C), Ser-Asp rich fibrinogen-binding protein genes (*sdr*G and *sdr*H), fibronectin-binding proteins (*pav*A), D-alanine-polyphosphoribitol ligase (*dlt*A), streptococcal plasmin receptor/GAPDH gene (*plr/gap*A), and clumping factor A gene (*clf*A). The autolysin gene (*atl*), which encodes the protein that exhibits vitronectin-binding activity, is involved in the primary attachment of *S. epidermidis* to a polystyrene surface and is similar to the major autolysin of *S. aureus* [18]. The *atl* gene was detected in all but 2 of the 18 screened isolates, which is important considering their varying adherence characteristics. 

The sequence type ST2, usually reported in hospital environments, is distributed around the globe [33]. The ST2 isolates in this study harboured the icaA gene and IS256, which have been associated with increased pathogenicity [34]. Similarly, both ST54 isolates possessed the *ica* genes and IS256. However, the sample size is not large enough to draw conclusive links between STs, biofilm-associated genes, ward types, and pathogenicity. Future studies involving *ica* gene expression in IS256 isolates will help delineate their specific relation. 

The detection of genetic determinants of biofilm formation in screened isolates in this study suggests that the infections caused by these strains will most likely be biofilm-related under favourable conditions, thus presenting treatment challenges. Among the chronically ill, the long-term hospitalised, and those harbouring invasive medical devices, biofilm-forming CoNS can be particularly problematic [1]. Again, since CoNS are responsible for most foreign body-related infections among individuals with temporarily or permanently implanted devices, the biofilm-forming potential of these isolates can facilitate their persistence at the local site or even in systemic circulation when they spread [2]. Currently, no antimicrobials specifically target bacteria growing in biofilms, leading to poor treatment outcomes [35]. Thus, it is essential to understand the mechanisms of biofilm formation to prevent them from forming. It is also critical to develop compounds that prevent or break down biofilms. However, the study was limited by a lack of clinical and in-depth demographic data that precluded the analysis of relationships between CoNS species, wards/subjects, and genomic profiles. Furthermore, the low number of sequenced isolates makes investigating the effect of gene presence on clinical outcomes challenging, and thus further studies are recommended.

## 5. Conclusions

The study provided insight into the biofilm-forming characteristics of coagulase-negative staphylococci isolated from the clinical setting. Isolates showed varying biofilm-forming capacities ranging from weak to strong, while others did not show the ability to form biofilms. *S. epidermidis*, *S. hominis* ssp. *hominis*, and *S. haemolyticus* were the most abundant species of strong biofilm formers. In addition, isolates showed a strong genetic basis for biofilm formation, as shown by the frequent detection of *ica*A, *ica*B, *ica*C, and *atl* genes, increasing our understanding of the phenotypic biofilm observation.

## Figures and Tables

**Table 1:** Categories of biofilm formation according to the CoNS species obtained.

Item	Strongly Adherent (%)	Moderate (%)	Weak (%)	Total (%)
*S. epidermidis*	3 (3.4)	8 (8.9)	5 (5.6)	16 (17.9)
*S. hominis* ssp. *hominis*	2 (2.2)	10 (11.2)	2 (2.2)	14 (15.7)
*S. sciuri*		5 (5.6)		5 (5.6)
*S. lentus*	1 (1.1)	9 (10.1)	3 (3.4)	13 (14.6)
*S. saprophyticus*		1 (1.1)	2 (2.2)	3 (3.4)
*S. gallinarum*		1 (1.1)	1 (1.1)	2 (2.2)
*S. capitis*		2 (2.2)		2 (2.2)
*S. lugdunensis*		2 (2.2)		2 (2.2)
*S. auricularis*		1 (1.1)		1 (1.1)
*S. xylosus*		5 (5.6)		5 (5.6)
*S. arlettae*	1 (1.1)			1 (1.1)
*S. hominis*		4 (4.5)		4 (4.5)
*S. succinus*	1 (1.1)	2 (2.2)	1 (1.1)	4 (4.5)
*S. haemolyticus*	2 (2.2)	11 (12.3)	2 (2.2)	15 (16.9)
*S. warneri*	1 (1.1)			1 (1.1)

**Table 2:** Biofilm/adherence-related genes found in isolates.

Isolate	Ward	CoNS Species	Adherence/Biofilm-Associated Genes	MLST	Insertion Sequence IS256
C7	3N ICU	*S. haemolyticus*	*atl,ebp*	ST3	+
C31	A1 Paediatric	*S. haemolyticus*	*atl, ebp*	Unknown	−
C35	E1 Paediatric	*S. epidermidis*	*atl, ebh, ebp, sdrE, sdrH, prgB/asc10, dltA, ebpC, pavA, flmH, slrA, plr/gapA, fsrA, fsrB, fsrC*	Unknown	+
C36	Neonatal ICU	*S. epidermidis*	*atl, ebh, ebp, icaA, icaB, icaC, icaR,*	ST54	+
C38	H2 Medical	*S. epidermidis*	*atl, ebh, ebp, icaA, icaB, icaC, icaR, sdrC, sdrG, sdrH, prgB/asc10, dltA, ebpC, pavA, slrA, fsrA, fsrB, fsrC*	ST83	+
C40	3N Extension	*S. epidermidis*	*atl, ebh, clfA, ebp, icaA, icaB, icaC, icaR, sdrG, sdrH, prgB/asc10, dltA, ebpC, pavA, slrA, plr/gapA*	ST54	+
C68	7F Paediatric	*S. epidermidis*	*atl, ebh, ebp, sdrH, flmH*	ST210	−
C81	F2 Surgical ward	*S. epidermidis*	*atl, ebh, ebp, icaA, icaB, icaC, icaR, sdrG, sdrH, asa1, dltA, ebpC, fss3, pavA, slrA, plr/gapA*	ST2	+
C119	2F Paediatric ICU	*S. epidermidis*	*sdrH*	Unknown	+
C122	Paediatric OPD	*S. epidermidis*	*atl, ebh, ebp, sdrG, sdrH, hcpB, htpB, orfH, flmH, nueA, tapT, fimC, fimD, fimD, pilU, pilQ, adeG, pgaC*	ST59	−
C127	Paediatric OPD	*S. epidermidis*	*atl, ebh, ebp, sdrG, sdrH, hcpB, flmH, nueA, fimC, fimD, pilU, pilQ, pgaC*	ST59	−
C133	Paediatric OPD	*S. epidermidis*	*atl, ebh, ebp, icaA, icaB, icaC, icaR, sdrC, sdrH*	ST490	−
C135	Paediatric OPD	*S. epidermidis*	*atl, ebp, icaA, icaB, icaC, icaR, sdrH*	Unknown	−
C137	Ward O	*S. epidermidis*	*ebp, icaA, icaB, sdrF, sdrH, hcpB, htpB, orfH, flmH, nueA, tapT, fimA, fimC, fimD, pilU, pilQ, adeG, pgaC*	Unknown	+
C138	H Ward	*S. epidermidis*	*atl, ebh, ebp, icaA, icaB, icaC, icaR, sdrH*	Unknown	−
C145	Casualty	*S. epidermidis*	*atl, ebh, ebp, icaA, icaB, icaC, icaR, sdrG, sdrH,*	ST2	+
C146	Paediatric	*S. epidermidis*	*atl, ebh, ebp, sdrG, sdrH, csgG, ecpA, fleR, fliQ, hcpB, htpB, orfH, flgC, flgC, plr/gapA, pilW, pgaC*	ST640	−
C148	Paediatric	*S. epidermidis*	*atl, ebp*	Unknown	+

## Data Availability

All data have been included in the manuscript and its associated Supplementary Materials.

## Supplementary Materials

The following supporting information can be downloaded at: https://www.mdpi.com/article/10.3390/genes14010104/s1, Figure S1: Colony colours of isolates on CRA. (A) Black colonies indicative of strong biofilm formation, (B) brown colonies indicative of moderate biofilm formation, and (C) red colonies indicating no biofilm formation.; Table S1: CoNS species distribution and antibiotic resistance profile of CoNS isolates; Table S2: CRA characteristics and classification of biofilm-forming capacities of CoNS isolates using different formulas.
