# Supplementary material for: Phenotypic and Genomic Insights into Biofilm Formation in Antibiotic-Resistant Clinical Coagulase-Negative Staphylococcus Species from South Africa
Source: Genes (Basel). 2022 Dec 29;14(1):104. doi: 10.3390/genes14010104 (PMC9858754; doi:10.3390/genes14010104)
Supplement: Supplementary file 1 [file genes-14-00104-s001.zip › genes-2087810-supplementary.pdf]

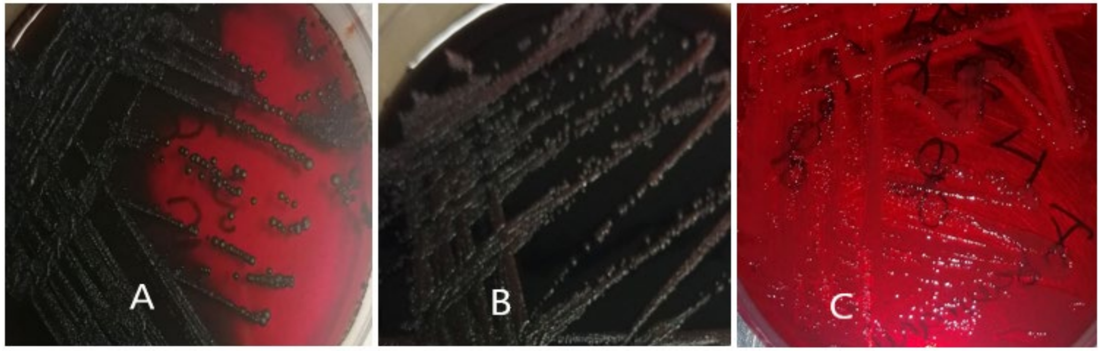

Figure S1. Colony colours of isolates on CRA. (**A**) Black colonies indicative of strong biofilm formation, (**B**) brown colonies indicative of moderate biofilm formation, and (**C**) red colonies indicating no biofilm formation.

Table S1. CoNS species distribution and antibiotic resistance profile of CoNS isolates.

Isolate ID	CoNS species	Ward type	OD_{570nm}	FOX	P	CPT	CIP	MXF	AZM	E	CN	AK	C	TE	DO	TEC	TGC	LZD	DA	RD	SXT	F
C1	*S. epidermidis*	Paediatric OPD	0.163		R																R	
C3	*S. epidermidis*	Ward & nursery	0.142	R	R		R	R	R	R	R								R		R	
C4	*S. epidermidis*	A1 paediatric ward	0.302	R	R		R	R	R	R			R						R	R	R	
C5	*S. hominis ssp hominis*	Paediatric OPD	0.281		R				R	R			R						R		R	
C6	*S. epidermidis*	Emergency department	0.2		R	R							R							R		R
C7	*S. haemolyticus*	3N ICU	0.145	R	R		R	R	R	R									R	R		
C9	*S. epidermidis*	-	0.175	R	R		R	R	R	R	R				R					R	R	
C10	*S. haemolyticus*	ICU	0.167	R	R		R	R	R	R		R							R	R		

C11	*S. hominis ssp hominis*	Casualty	0.197		R															R	
C13	*S. hominis ssp hominis*	Neonatal ICU	0.264	R	R		R	R	R	R		R	R	R	R	R	R	R	R	R	R
C17	*S. hominis ssp hominis*	H1 Medical ward	0.343	R	R			R	R	R		R						R			
C18	*S. haemolyticus*	KMMC Clinic	0.31		R				R	R										R	
C19	*S. epidermidis*	Neonatal ICU	0.347	R	R				R	R										R	
C20	*S. hominis ssp hominis*	H2 Medical ward	0.352		R	R		R				R	R	R	R	R	R		R	R	R
C21	*S. sciuri*	ICU	0.356	R	R		R	R	R	R								R	R	R	
C22	*S. lentus*	Ward D	0.226	R	R		R	R	R	R			R	R					R	R	
C23	*S. lentus*	Paediatric OPD	0.177		R																
C26	*S. lentus*	-	0.207	R	R																
C27	*S. haemolyticus*	Ward D	0.224	R	R						R	R	R	R	R	R	R		R	R	R
C31	*S. haemolyticus*	A1 Paediatric ward	0.292	R	R				R	R											
C32	*S. sciuri*	E2 Paediatric ICU	0.407	R	R		R	R	R	R								R	R		
C35	*S. saprophyticus*	E1 Paediatric ward	0.194	R	R	R	R	R	R	R		R	R	R				R		R	
C36	*S. saprophyticus*	Neonatal ICU	0.179	R	R		R		R	R		R	R	R	R	R	R		R	R	R
C37	*S. lentus*	Neonatal ICU	0.276		R		R	R	R	R		R						R	R	R	
C38	*S. epidermidis*	H2 Medical ward	0.21	R	R		R	R	R	R										R	
C39	*S. lentus*	5B2 Medical ward	0.279	R	R		R	R	R	R		R	R	R				R	R	R	

C40	*S. hominis ssp hominis*	3N Extension ward	0.312	R	R			R	R	R							R
C42	*S. haemolyticus*	5F Medical ward	0.299	R	R		R	R	R	R			R	R		R	R
C43	*S. lentus*	D1 Medical ward	0.271	R	R		R	R	R	R	R	R			R		R
C44	*S. gallinarum*	W E	0.168	R	R												
C47	*S. hominis ssp hominis*	D1 Medical ward	0.271	R	R				R	R							R
C48	*S. hominis ssp hominis*	3F Obstetrics & Gynaecology ward	0.178	R	R				R	R							R
C49	*S. capitis*	3N Extension ward	0.209	R	R		R	R	R	R			R		R	R	
C53	*S. lentus*	3N Main	0.297	R	R				R	R					R	R	R
C54	*S. haemolyticus*	3N Extension ward	0.333	R	R		R	R	R	R					R	R	
C55	*S. lentus*	Ward & Nursery	0.293		R				R	R							
C56	*S. lentus*	E1 Paediatric ward	0.246	R	R	R		R	R	R			R	R	R	R	R
C57	*S. epidermidis*	7B2 Extension ward	0.322	R	R	R	R	R	R	R	R	R			R	R	R
C58	*S. epidermidis*	Surgical OPD	0.289	R	R		R	R	R	R		R	R	R	R	R	R
C61	*S. haemolyticus*	D1 Medical ward	0.226	R	R		R	R	R							R	R
C66	*S. lentus*	Paediatric OPD	0.21		R	R											R

C68	*S. epidermidis*	7F Paediatric ward	0.174	R	R				R	R	R	R	R				R
C72	*S. lentus*	2R Surgical ICU	0.195		R												
C73	*S. lentus*	Ward F	0.235		R												R
C74	*S. epidermidis*	Paediatric OPD	0.398		R			R									R
C75	*S. lugdunensis*	-	0.26		R												
C81	*S. hominis ssp hominis*	F2 Surgical ward	0.251	R	R		R	R	R	R					R	R	R
C87	*S. auricularis*	3N Main	0.369	R	R				R	R		R			R		
C93	*S. lentus*	H1 Medical ward	0.468	R	R		R	R	R	R	R				R	R	R
C100	*S. haemolyticus*	Emergency department	0.346	R	R				R	R							R
C102	*S. haemolyticus*	Paediatric OPD	0.278	R	R		R	R	R	R		R	R		R	R	R
C104	*S. capitis*	7F Paediatric ward	0.37		R		R	R	R	R		R			R	R	R
C105	*S. hominis ssp hominis*	Paediatric OPD	0.315	R	R		R		R	R		R	R			R	R
C107	*S. lugdunensis*	-	0.367		R												
C110	*S. xylosus*	H2 Medical ward	0.278	R	R							R	R	R		R	
C113	*S. xylosus*	Medical OPD	0.336	R	R				R	R						R	
C114	*S. sciuri*	Medical OPD	0.362		R				R	R							
C116	*S. hominis ssp hominis*	Paediatric OPD	0.469		R				R	R							R
C118	*S. hominis ssp hominis*	Ward D	0.432	R	R	R			R	R		R	R				R

C119	*S. xylosus*	2F Paediatric ICU	0.34	R	R	R			R	R		R	R			R
C120	*S. gallinarum*	Paediatric OPD	0.327	R	R				R	R	R					R
C121	*S. hominis ssp hominis*	E1 Paediatric ward	0.308	R	R											R
C122	*S. hominis ssp hominis*	Paediatric OPD	0.233	R	R											R
C123	*S. haemolyticus*	Emergengy And Accident Unit	0.318		R				R	R						
C125	*S. arlettae*	KMMC Clinic	0.701	R	R				R	R	R			R	R	R
C126	*S. epidermidis*	1F Male surgical Ward	0.437		R											
C127	*S. hominis*	1F Male Surgical ward	0.298	R	R				R	R						R
C128	*S. hominis*	1F Male surgical Ward	0.394	R	R		R	R	R	R				R		R
C129	*S. haemolyticus*	1F Male Surgical ward	0.464	R	R		R	R	R	R				R	R	
C131	*S. sciuri*	Emergengy And Accident Unit	0.337	R	R		R		R	R		R	R			R
C132	*S. sciuri*	C2 Surgical ward	0.364	R	R	R	R		R	R				R	R	R
C133	*S. epidermidis*	Paediatric OPD	0.498	R	R											R
C134	*S. epidermidis*	Casualty	0.364	R	R		R		R	R		R	R			

C135	*S. saprophyticus*	Paediatric OPD	0.302	R	R	R	R	R	R	R	R	R				R
C136	*S. succinus*	H Ward	0.205		R			R	R							R
C137	*S. epidermidis*	Ward O	0.083	R	R	R		R	R					R		R
C138	*S. hominis*	H Ward	0.279	R	R											R
C139	*S. succinus*	3N Extension ward	0.362	R	R			R	R	R				R	R	R
C141	*S. haemolyticus*	3N Extension ward	0.538	R	R	R	R		R						R	
C142	*S. succinus*	Paediatric OPD	0.497	R	R											R
C143	*S. warneri*	3N ICU	0.426	R	R			R	R				R		R	R
C144	*S. epidermidis*	3N Extension ward	0.564	R	R	R	R	R	R	R				R	R	R
C145	*S. epidermidis*	Casualty	0.373	R	R			R	R				R			
C147	*S. xylosus*	Ward and Nursery	0.215	R	R	R	R	R	R	R				R		R
C148	*S. xylosus*	D5 Ward	0.252	R	R			R	R							
C149	*S. hominis*	KMMC Clinic	0.24	R	R			R	R							R
C150	*S. succinus*	Paediatric OPD	0.293	R	R			R	R	R	R	R				R
C151	*S. haemolyticus*	E1 Paediatric Ward	0.334	R	R	R	R	R	R						R	
C152	*S. haemolyticus*	ICU	0.284	R	R	R	R								R	

Abbreviations: FOX, cefoxitin; P, penicillin G; CPT, ceftaroline; CIP, ciprofloxacin; MXF, moxifloxacin; AZM, azithromycin; E, erythromycin; CN, gentamicin; AK, amikacin; C, chloramphenicol; TE, tetracycline; DO, doxycycline; TEC, teicoplanin; TGC, tigecycline; LZD, linezolid; DA, clindamycin; RD, rifampicin; SXT, sulphamethoxazole/trimethoprim; F, nitrofurantoin; R, resistant; OPD, Outpatient Department; ICU, Intensive Care Unit; CRA, Congo Red agar

Black colonies were considered positive for biofilm formation on CRA, brown colonies; pink/red colonies were negative for biofilm formation on CRA while brown colonies on CRA were considered moderate biofilm formers

Table S2. CRA characteristics and classification of biofilm-forming capacities of CoNS isolates using different formulas.

Isolates ID	CoNS species	CRA characteristics	OD≤ODc (negative)	ODc<OD≤(2×ODc) (weak)	(2×ODc)<OD≤(4×ODC) (moderate)	(4×ODc)<OD (strong)	BF = AB-CW				BF=AB/CW			
							negative	weak	moderate	Strong	negative	weak	moderate	strong
C1	*S. epidermidis*	Black		√			√				√			
C3	*S. epidermidis*	Black		√			√				√			
C4	*S. epidermidis*	Black			√			√				√		
C5	*S. hominis ssp hominis*	Black			√			√				√		
C6	*S. epidermidis*	Black		√			√				√			
C7	*S. haemolyticus*	Brown		√			√				√			
C9	*S. epidermidis*	Black		√			√				√			
C10	*S. haemolyticus*	Brown		√			√				√			
C11	*S. hominis ssp hominis*	Black		√			√				√			
C13	*S. hominis ssp hominis*	Black			√			√				√		
C17	*S. hominis ssp hominis*	Brown			√				√			√		
C18	*S. haemolyticus*	Brown			√				√			√		
C19	*S. epidermidis*	Black			√				√			√		
C20	*S. hominis ssp hominis*	Brown			√				√			√		
C21	*S. sciuri*	Black			√				√			√		
C22	*S. lentus*	Brown			√			√				√		
C23	*S. lentus*	Brown		√			√				√			
C26	*S. lentus*	Brown		√				√			√			
C27	*S. haemolyticus*	Brown			√			√				√		
C31	*S. haemolyticus*	Brown			√			√				√		
C32	*S. sciuri*	Brown			√					√		√		
C35	*S. saprophyticus*	Brown		√			√				√			
C36	*S. saprophyticus*	Black		√			√				√			
C37	*S. lentus*	Red			√			√				√		
C38	*S. epidermidis*	Black			√			√				√		
C39	*S. lentus*	Brown			√			√				√		

C40	*S. hominis ssp hominis*	Black		√				√			√	
C42	*S. haemolyticus*	Brown		√			√				√	
C43	*S. lentus*	Black		√			√				√	
C44	*S. gallinarum*	Black	√			√				√		
C47	*S. hominis ssp hominis*	Brown		√			√				√	
C48	*S. hominis ssp hominis*	Brown	√			√				√		
C49	*S. capitis*	Brown		√			√				√	
C53	*S. lentus*	Brown		√			√				√	
C54	*S. haemolyticus*	Red		√				√			√	
C55	*S. lentus*	Brown		√			√				√	
C56	*S. lentus*	Brown		√			√				√	
C57	*S. epidermidis*	Black		√				√			√	
C58	*S. epidermidis*	Black		√			√				√	
C61	*S. haemolyticus*	Brown		√			√				√	
C66	*S. lentus*	Brown		√			√				√	
C68	*S. epidermidis*	Brown	√			√				√		
C72	*S. lentus*	Brown	√			√				√		
C73	*S. lentus*	Black		√			√				√	
C74	*S. epidermidis*	Brown		√				√			√	
C75	*S. lugdunensis*	Brown		√			√				√	
C81	*S. hominis ssp hominis*	Black		√			√				√	
C87	*S. auricularis*	Black		√				√			√	
C93	*S. lentus*	Red			√				√			√
C100	*S. haemolyticus*	Brown		√			√				√	
C102	*S. haemolyticus*	Brown		√			√				√	
C104	*S. capitis*	Black		√				√			√	
C105	*S. hominis ssp hominis*	Black		√			√				√	
C107	*S. lugdunensis*	Black		√				√			√	
C110	*S. xylosus*	Brown		√			√				√	
C113	*S. xylosus*	Black		√			√				√	
C114	*S. sciuri*	Black		√				√			√	

C116	*S. hominis ssp hominis*	Black				✓				✓			✓	
C118	*S. hominis ssp hominis*	Brown				✓			✓				✓	
C119	*S. xylosus*	Black			✓			✓				✓		
C120	*S. gallinarum*	Brown			✓			✓				✓		
C121	*S. hominis ssp hominis*	Black			✓			✓				✓		
C122	*S. hominis ssp hominis*	Brown			✓		✓					✓		
C123	*S. haemolyticus*	Brown			✓			✓				✓		
C125	*S. arlettae*	Black				✓				✓				✓
C126	*S. epidermidis*	Brown				✓			✓				✓	
C127	*S. hominis*	Brown			✓			✓				✓		
C128	*S. hominis*	Brown			✓				✓			✓		
C129	*S. haemolyticus*	Brown				✓				✓			✓	
C131	*S. sciuri*	Brown			✓			✓				✓		
C132	*S. sciuri*	Brown			✓				✓			✓		
C133	*S. epidermidis*	Black				✓				✓			✓	
C134	*S. epidermidis*	Brown			✓				✓			✓		
C135	*S. saprophyticus*	Brown			✓			✓				✓		
C136	*S. succinus*	Brown		✓			✓				✓			
C137	*S. epidermidis*	No growth	✓				✓				✓			
C138	*S. hominis*	Brown			✓			✓				✓		
C139	*S. succinus*	Black			✓				✓			✓		
C141	*S. haemolyticus*	Brown				✓				✓			✓	
C142	*S. succinus*	Black				✓				✓			✓	
C143	*S. warneri*	Brown				✓			✓				✓	
C144	*S. epidermidis*	Black				✓				✓			✓	
C145	*S. epidermidis*	Brown			✓				✓			✓		
C147	*S. xylosus*	Black			✓		✓					✓		
C148	*S. xylosus*	Brown			✓			✓				✓		
C149	*S. hominis*	Black			✓		✓					✓		
C150	*S. succinus*	Brown			✓			✓				✓		
C151	*S. haemolyticus*	Brown			✓			✓				✓		

C152	*S. haemolyticus*	Brown	√		√		√	
ATCC 35984	*S. epidermidis*			√		√		√
